# Simple Urea Immersion Enhanced Removal of Tetracycline from Water by Polystyrene Microspheres

**DOI:** 10.3390/ijerph15071524

**Published:** 2018-07-19

**Authors:** Junjun Ma, Bing Li, Lincheng Zhou, Yin Zhu, Ji Li, Yong Qiu

**Affiliations:** 1 State Key Joint Laboratory of Environment Simulation and Pollution Control, School of Environment, Tsinghua University, Beijing 100084, China; mjj16@mails.tsinghua.edu.cn; 2 School of Energy and Environmental Engineering, University of Science and Technology Beijing, Beijing 100083, China; libing@ustb.edu.cn; 3 State Key Laboratory of Applied Organic Chemistry, College of Chemistry and Chemical Engineering, Institute of Biochemical Engineering and Environmental Technology, Lanzhou University, Lanzhou 730000, China; zhoulc@lzu.edu.cn; 4 School of Environmental and Civil Engineering, Jiangnan University, Wuxi 214122, China; zyfreely@vip.jiangnan.edu.cn (Y.Z.); liji@jiangnan.edu.cn (J.L.)

**Keywords:** microsphere resin, urea immersion, adsorption isotherms, surface characterization, kinetics analysis, multilayer adsorption

## Abstract

Antibiotics pose potential ecological risks in the water environment, necessitating their effective removal by reliable technologies. Adsorption is a conventional process to remove such chemicals from water without byproducts. However, finding cheap adsorbents with satisfactory performance is still a challenge. In this study, polystyrene microspheres (PSM) were enhanced to adsorb tetracycline by surface modification. Simple urea immersion was used to prepare urea-immersed PSM (UPSM), of which surface groups were characterized by instruments to confirm the effect of immersion. Tetracycline hydrochloride (TC) and doxycycline (DC) were used as typical adsorbates. The adsorptive isotherms were interpreted by Langmuir, Freundlich, and Tempkin models. After urea immersion, the maximum adsorption capacity of UPSM at 293 K and pH 6.8 increased about 30% and 60%, achieving 460 mg/g for TC and 430 mg/g for DC. The kinetic data were fitted by first-order and second-order kinetics and Weber–Morris models. The first-order rate constant for TC adsorption on UPSM was 0.41 /h, and for DC was 0.33 /h. The cyclic urea immersion enabled multilayer adsorption, which increased the adsorption capacities of TC on UPSM by two to three times. The adsorption mechanism was possibly determined by the molecular interaction including π–π forces, cation-π bonding, and hydrogen bonding. The simple surface modification was helpful in enhancing the removal of antibiotics from wastewater with similar structures.

## 1. Introduction

Antibiotics are a concern as contaminants in the water environment due to their potential ecological risks and ubiquitous distribution in the world [1]. Among the many categories of antibiotics, reagents in the tetracycline group have been used extensively to control disease in human beings and livestock, due to their broad-spectrum antimicrobial activities [2]. Tetracycline (TC) and doxycycline (DC) are typical widely used tetracycline antibiotics. The potential health risks of residual antibiotics in the aqueous environment lie in the possible development of drug resistance in bacteria, challenging the current therapies of known antibiotics [3]. Compared with aerosol and soil, aqueous phase transporting in wastewater, sewers, surface water, and groundwater was thought to be the dominant way to spread antibiotics and antibiotic resistance to remote areas and society [4]. Therefore, antibiotic contamination is a serious environmental issue that needs an effective response.

Many effective techniques have been developed to remove or degrade residual antibiotics in wastewater, such as adsorption [5], advantage oxidation [6,7], activated sludge transformation [8], electrochemical treatment [9], biological composition [10], membrane filtration [11], etc. Adsorption was an old but clean, easy, and efficient process to remove aqueous pollutants [12]. Engineers could adapt the adsorption process rapidly by utilizing existing apparatus such as filter or dosing pumps. Thus large wastewater treatment plants in the pharmaceutical industry could be easily equipped, or on-site small apparatuses could be used in rural areas. During adsorption treatment, very few byproducts are produced and released into the water, resulting in less risk of unknown and uncontrolled products in oxidation processes that could be hazardous to the ecosystem. There were many difficulties in applying adsorption in antibiotics removal, such as unselective adsorption, insufficient capacity, and high cost of adsorbent. Finding cheap adsorbent with satisfactory performance is still a challenge. Thus people have attempted to use carbonaceous materials [13], sludge [14], natural minerals [15], siliceous materials [16,17], and polymer resins [18,19,20] to remove antibiotics. Therefore, understanding the adsorption properties of antibiotics is important for engineering approaches to improve their performance.

Polymer resin microspheres, with the merits of low cost, high porosity, large surface area, and adsorption capacity, have potential for commercial application [14,19,20,21,22]. Surface modification is important to acquire designed functions of adsorbents. Surface modification was a typical way to improve the selectivity and capacity of antibiotics adsorption [22,23], as well as easier phase separation by magnetic forces [14,19,21]. Modification of the functional groups on the surface can increase adsorption capacity by enhancing the chemical bonds to the target chemicals. For example, adsorption of TC has been enhanced by surface functionalization by the amino-ferrous group [24] and amino-copper group [25]. Modification with the anion exchange group improved adsorption capacity to more than 355 mg/g [26].

Tetracycline reagents have multiple functional groups (Supplementary Figure S1), including phenol, amino, alcohol, and ketone groups, which are capable of electronic coupling and various reactions. One idea is to impregnate an amino group (–NH_2_) on the adsorbent surface as a neutral anion receptor to link an ester group (–COO–R) in antibiotic molecules, in order to form chelated complexes with hydrogen bond acceptors like ditopic carboxylates. Another choice is to use a carbonyl group as an electron donor to enhance the π–π interaction and cation-π bonding. As a kind of simple and cheap commercial material, urea (CO(NH_2_)_2_) combined with two amino (–NH_2_) and one carbonyl (C=O) functional group, makes it a good agent to interact with tetracycline molecules.

In this paper, we modified the surface of polystyrene ethylenediaminetetraacetate (EDTA) microsphere (PSM) resin by simply immersing it in urea solution. Then, the urea modified PSM (UPSM) was characterized by Brunauer–Emmett–Teller (BET), x-ray photoelectron spectroscopy (XPS), and infrared (IR) to confirm the effects of impregnation. Later, we conducted adsorption experiments to evaluate their capacity to remove TC and DC from water. Finally, the interaction between antibiotic molecules and UPSM surface is discussed.

## 2. Materials and Methods

### 2.1. Microsphere Modification

Polystyrene EDTA microsphere (PSM) resins were synthesized according to a reported thermal-solvent method [26]. The polystyrene microspheres were first synthesized by gentle agitation to control the average diameter to about 900 μm. Then the surface was functionalized by adding EDTA groups by 2 steps of reactions. The formula of PSM is shown in Supplementary Figure S1. The material has been successfully applied to treat aqueous pollutants [27,28].

The urea-immersed PSM (UPSM) was acquired by immersing the PSM into 100 g/L of urea solution for 12 h. The urea solution was prepared by dissolving 5 g urea powder (Alfa Aesar, Johnson Matthey Company, West Chester, PA, USA) in 50 mL of ultrapure water. Then 2 g of PSM was mixed with 50 mL of urea solution in a 100 mL 3-necked glass flask. The mixture was agitated in a thermostatic shaker at 150 rpm and 25 ± 1 °C for 12 h. After filtration, the UPSM were intensively washed by ultrapure water 10 times to remove the excessive urea. Then the UPSM were dried in an oven with desiccants at room temperature overnight.

### 2.2. Surface Characterization

The surface morphologies of PSM and UPSM were characterized by using a scanning electron microscope (SEM; HT 7700, Hitachi Corp., Tokyo, Japan). The SEM images were similar between PSM and UPSM, as shown in Supplementary Figure S2. Their surface areas in Brunauer–Emmett–Teller (BET) were determined by N_2_ adsorption-desorption isotherm at liquid nitrogen temperature (TriStar 3020 II, Micromeritics Instrument Corp., Norcross, GA, USA). The instrumental parameters were default as described in the manual and guidance. The surface area of UPSM was slightly higher than that of PSM, as shown in Supplementary Figure S3. The pore size distributions of PSM and UPSM were close to each other (Supplementary Figure S4). 

The surface functional groups of PSM and UPSM were identified by Fourier transform infrared (FT-IR) spectroscopy using the KBr tableting technique on an FT-IR spectrometer (PE Spectrum GX, Perkin-Elmer Corp., Waltham, MA, USA) in transmission mode. The elemental analysis was conducted by x-ray photoelectron spectroscopy (XPS) on an electron spectrometer (PHI Quantera II, Ulvac-Phi Corp., Chigasaki, Japan) using 300 WAl-Ka radiation.

### 2.3. Chemicals and Analysis

The target antibiotic reagents, tetracycline hydrochloride (TC) and doxycycline hydrochloride (DC), were purchased from a commercial supplier (Inalco spa Milano, Milan, Italy). The molecular structures and other information of urea and tetracycline are shown in Supplementary Table S1. All chemicals were of analytical grade and used without pretreatment. Ultrapure water (Milli-Q, Millipore Co., Burlington, MA, USA) was used to prepare the solutions.

The concentrations of TC and DC in aqueous solution were determined on a UV-Vis spectrophotometer (U-3900, Hitachi Corp., Japan). The specific wavelength for TC was 360 nm and for DC was 325 nm according to the maximum absorption. The concentration was calibrated from linear standard curves (R^2^ > 0.999). Control experiments were conducted by mixing 40 mg/L tetracycline with 100 g/L urea with equal volume. After 12 h, the residual concentrations of TC and DC changed by less 1% of the initial values, indicating that tetracycline molecules are inert with urea in the solution.

Hydrochloric acid (HCl) and sodium hydroxide (NaOH) were ordered from a local supplier (Shanghai Chemical Reagents Co., Shanghai, China) to prepare HCl solution in 0.1 mol/L (M) and NaOH in 0.1 M. The bulk pH of the solutions was adjusted before adsorption experiments by dosing drops of above acid and base manually. The instant pH value was read out by a laboratory pH meter 1 min after dosing.

### 2.4. Adsorption Experiments

Preliminary adsorption experiments were conducted to determine the optimal bulk pH in the gradient range (pH 2.7, 6.8, 8.5 and 10.3). Kinetic curves at the gradient pH were acquired by exposing 60 mg/L of TC or DC to 60 mg of UPSM. The results are shown in Supplementary Figure S5, in which the optimal pH was determined to be 6.8.

For isothermal experiments, tetracycline solutions at 16 levels of initial concentration (100–2000 mg/L) were prepared to expose to 60 mg of PSM or UPSM in a volume of 20 mL. The mixture was agitated in a thermostatic shaker at a speed of 150 rpm and temperature of 25 °C. The experiments lasted for 24 h to ensure that the adsorption reached the equilibrium state.

For kinetic experiments, 60 mg of PSM or UPSM was dosed into tetracycline solutions at 5 levels of initial concentration (100–300 mg/L). The experiments lasted for 100 min and the concentrations of TC and DC were determined to obtain the kinetics.

### 2.5. Recycling Adsorption by Urea Immersion

Recycling adsorption by UPSM was achieved by using urea immersion after the saturated adsorption of tetracycline. First, 40 mg/L of TC was adsorbed onto 60 mg of UPSM for 12 h. After that, the UPSM were rapidly washed by ultrapure water 10 times to remove urea. Second, the USPM were again immersed in 20 mL of urea solution at a concentration of 100 g/L for 12 h. Finally, the UPSM were cleaned and exposed to 40 mg/L of TC solution for an additional cycle of adsorption. In total there were 10 cycles of adsorption-immersion. The TC concentrations were analyzed for comparison with the control experiments using UPSM.

USPM after 10 cycles were further examined for the desorption property under thermal, acidic, and alkaline treatment. Thermal desorption was conducted at 35 °C by soaking the UPSM in 20 mL of pure water in a water bath for 12 h. The acidic desorption was conducted by immersing the UPSM in 20 mL of 0.1 M HCl for 3 h. The alkaline desorption was achieved under similar conditions. Control experiments were conducted in parallel. The experimental duration was 7 h. The thermal stability of tetracycline was examined in a water bath at 95 °C for 1 h.

### 2.6. Data Interpretation

We evaluated the solid surface loading (*q*_t_) of tetracycline onto microsphere resin by the following equation, as described in the literature [29]:

*q*_t_ = (*C*_0_ − *C*_t_)*·V*/*M* (1)

where *q*_t_ is the surface loading of adsorbates on adsorbents (mg/g), *M* is the mass of adsorbent (g), *V* is the volume of the solution (L), and *C*_0_ and *C*_t_ are bulk tetracycline concentrations initially and at time *t* of the experiment, respectively (mg/L). In the case of isothermal experiments, subscript *e* was used instead of *t* to represent the equilibrium state, as *q*_e_ and *C*_e_.

The isothermal models, including Langmuir, Freundlich, and Tempkin, are shown in Table 1. The kinetic models including first-order, second-order, and Weber–Morris models are also shown in the table. The parameters were estimated by using the linear Lineweaver–Burk equation (Excel 2010, Microsoft Corp., Redmond, WA, USA), and the correlation coefficients were used to evaluate the quality of data interpretation.

## 3. Results and Discussion

### 3.1. Solid Surface Characterization

#### 3.1.1. Surface Characterization

Similar SEM images of PSM and UPSM (Supplementary Figure S2) suggest that urea immersion did not physically modify the surface of the microspheres. The pore size distribution curves of UPSM and PSM were also similar to each other (Supplementary Figure S4), but the pore structures were slightly different (Supplementary Table S2). The average pore size of UPSM (20.4 nm) was 10% lower than that of PSM (22.6 nm) and pore volume (0.34 cm^3^/g) was 13% higher than PSM’s. Consequently, the BET surface area of UPSM (112.42 m^2^/g) was about 60% higher than that of PSM (71.69 m^2^/g), according to Supplementary Figure S3.

#### 3.1.2. FT-IR Analysis

**Figure 1:**
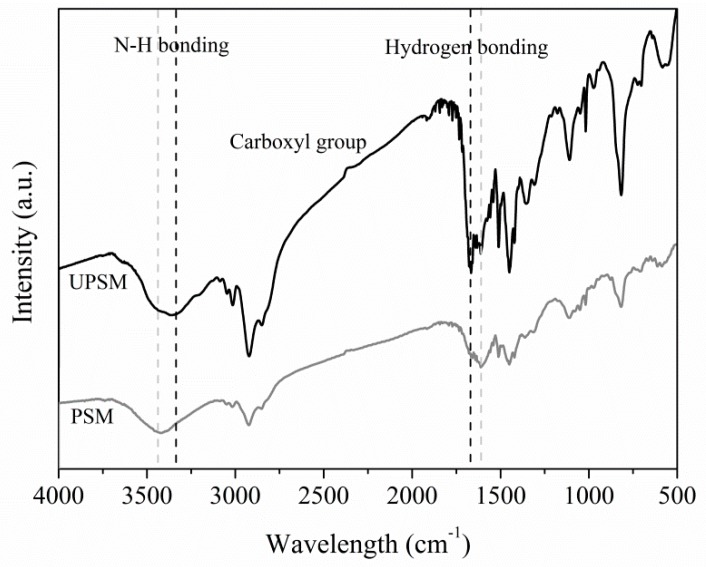
FT-IR spectra of fresh polystyrene microspheres (PSM) and urea-immersed PSM (UPSM).

The FT-IR spectra (Figure 1) suggest more amino and imino groups on UPSM than on PSM. Although both PSM and UPSM showed vibration of imino bond (N–H) in the amino/imino (–NH_2_/–NH) group in the range of 3300–3500 cm^−1^ [15], UPSM had a broader curve at about 3400 cm^−1^. Carbonyl groups in UPSM and PSM were slightly different, in the range of 2500–3400 cm^−1^. The broader band of UPSM at 1640–1650 cm^−1^ refers to the more singular hydrogen bonds between amino and carbonyl groups.

#### 3.1.3. XPS Analysis

**Figure 2:**
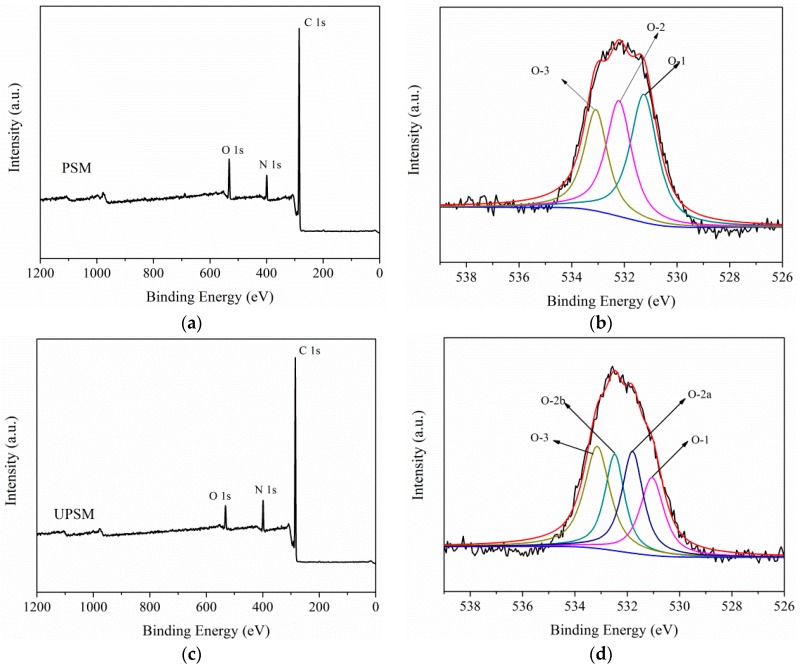
X-ray photoelectron spectroscopy (XPS) spectra of fresh PSM and UPSM. (**a**) Full spectrum of PSM; (**b**) O1s deconvolution of PSM; (**c**) full spectrum of PSM; (**d**) O1s deconvolution of UPSM.

XPS analysis (Figure 2) was used to confirm the variation of surface functional groups. PSM and UPSM had similar full XPS spectra (Figure 2a,c), as well as C 1s and N 1s deconvolution spectra (Supplementary Figure S6) because of their similar chemical components. PSM and UPSM had slightly different O 1s deconvolution spectra (Figure 2b,d). PSM showed subpeaks of C=O at 531.5 eV (O1), O–C–O at 532.5 eV (O2), and C–O at 533.1 eV (O3), while UPSM showed two small peaks at 532.7 eV (O2a) and 531.3 eV (O2a). The highest subpeak of PSM was 531.3 eV (O1) but that of UPSM shifted to 533.1 eV (O3). This shift might be related to the hydrogen bonding formed on the surface, such as H–N…H–C=O.

### 3.2. Adsorption Isotherms and Kinetics

#### 3.2.1. Isothermal Modeling

**Figure 3:**
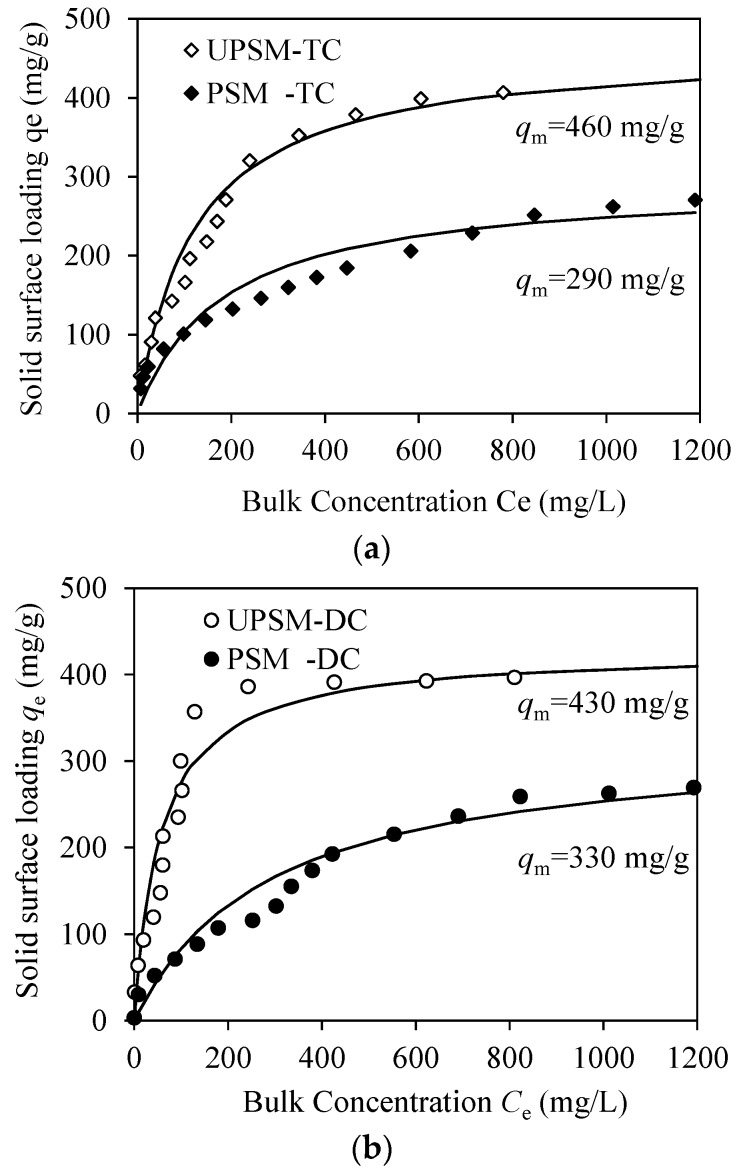
Adsorption isotherms of tetracycline antibiotics on microspheres. (**a**) TC on UPSM and PSM; (**b**) DC on UPSM and PSM. Observing data are fitted by the Langmuir model and the maximum adsorption capacity (*q*_m_) is shown near the curves.

The parameters of isothermal models were close for TC and DC, as shown in Table 2. According to the correlation coefficient (R^2^), the Langmuir and Freundlich models were not perfect to fit the data but better than the Tempkin model. The data-fitting results by the Langmuir model are shown in Figure 3 as an example. The maximum loading rate (*q*_m_) of TC on UPSM was 460 mg/g at pH 6.8 and 25 °C. This capacity was about 60% higher than that on PSM (290 mg/g). The adsorption capacity of DC on UPSM (430 mg/g) was 30% higher than that on PSM (330 mg/g). The higher adsorption capacity of UPSM than PSM was accordant to the larger BET surface area than PSM.

#### 3.2.2. Kinetics Modeling

**Figure 4:**
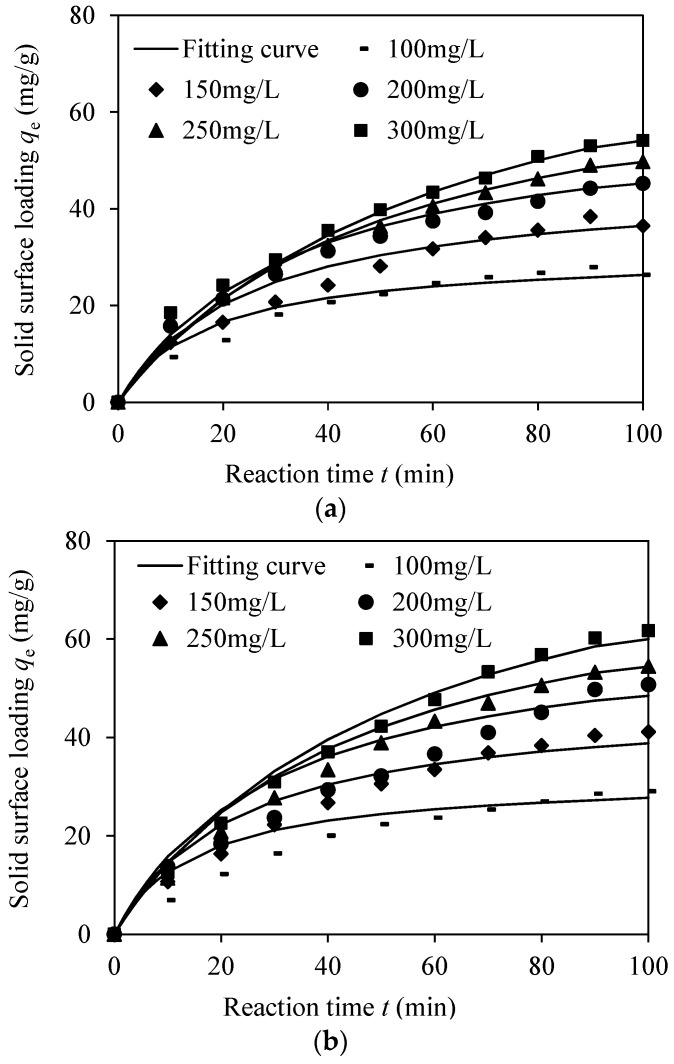
Adsorption kinetics of (**a**) TC and (**b**) DC on UPSM. The data fitting curves were printed in the same line type for each experiment.

The model parameters of the three kinetic models for tetracycline on UPSM are shown in Table 3. Similar to previous studies of microsphere adsorption [29,30], all three models were capable of explaining the kinetic data satisfactorily. As an example, the data curving fitting results obtained by the second-order kinetic model are shown in Figure 4. The performance of the global fitting was not as good as expected according to the correlation coefficient, partly because the initial concentrations showed small effects on the shape of the kinetic curves. The rate constants for each kinetic experiment showed a correlation to the initial concentrations as shown in Supplementary Table S3.

Kinetics is useful to determine the optimal volume of reactors to ensure certain removal efficiency. The duration of half reduction can be calculated as *t*_1/2_ = ln2/*K*_1_, where *K*_1_ is the rate constant of first-order kinetics. According to Table 3, the rate constant *K*_1_ for TC and DC adsorption is 0.33 and 0.41 /h, respectively, indicating that 50% removal of initial concentration occurred in 1.7 and 2.1 h. This duration covers the practical hydraulic retention time in filters.

#### 3.2.3. Adsorption Performance

Table 4 shows a comparison of adsorption capacities of UPSM in this study with other materials in the literature. Polymer resins showed a promising capacity to remove TC, especially after surface modification by graphene oxide, which reached 198 mg/g [31]. Using a fabricated nanosheet can increase the capacity to 315 mg/g [32]. The silicon-based material enhanced the capacity to 303 mg/g [33]. Activated carbon achieved satisfactory performance in removing TC [34]. In this study, the adsorption capacity of TC by UPSM (460 mg/g) was slightly higher than that of the above materials.

The magnetic particles on graphene oxide nanosheets showed impressively high adsorption capacity for tetracycline at 714 mg/g [35]. By comparison with UPSM, the material of the graphene oxide nanosheet is a little bit expensive and lacks commercialization. Additionally, structures of such material are not easy to integrate with existing filters in practice. UPSM in this study are cheap. Moreover, they are recyclable by heating to destroy the urea. What is most important is that UPSM can be easily used in conventional filters due to their physical size and hardness.

It is better to estimate adsorption performance in similar concentrations of adsorbates in the actual matrix. Tetracycline in a surface water environment, e.g., rivers and lakes, is generally at trace levels of several μg/L [1]. Its concentration in municipal wastewater might be as high as 150 μg/L [41]. The concentration levels in this study were selected to prove the concept and feasibility. Evaluating UPSM for tetracycline removal at trace levels is beyond the scope of this paper. Nevertheless, in the case of industrial wastewater treatment, when tetracycline concentration may exceed the mg/L level, the results in this study can be used as a reference.

### 3.3. Recycling Adsorption and Desorption

#### 3.3.1. Adsorption by Cycle Urea Immersion

**Figure 5:**
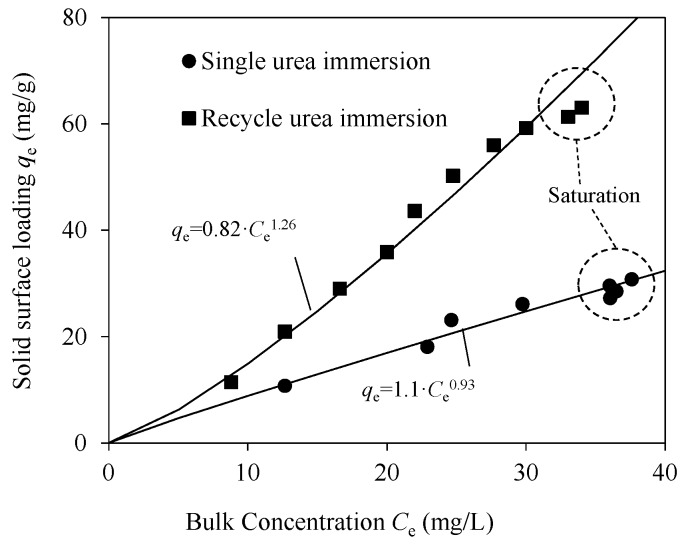
Comparison of isotherms of TC on UPSM with urea immersion in single and recycling modes. Experimental conditions include pH 6.8 and 25 °C for 12 h. Data is fitted by the Freundlich equation. The dashed circles highlight the saturation level of bulk concentration after repeated exposure of UPSM to TC solution.

The isotherms of TC on UPSM by single and cycle urea immersion are compared in Figure 5. The Freundlich equation was used to interpret the data. The values of parameter *n* for both modes (*n* = 0.93 and *n* = 1.26) were close to 1, indicating linear isotherm in low concentrations. In single immersion mode, the bulk concentration was saturated at about 37 mg/L after 5 cycles, while it took 10 cycles to saturate at about 35 mg/L in cycle immersion mode. This result suggests that multilayer adsorption increased the capacity but such effect reduced gradually with the increasing number of layers. At *C*_e_ = 20 mg/L, the adsorption capacity was twice that in single immersion mode. The improvement was three times at *C*_e_ = 30 mg/L. The cycle urea immersion obviously increased the adsorption capacity of UPSM by two to three times.

#### 3.3.2. Desorption Performance

**Figure 6:**
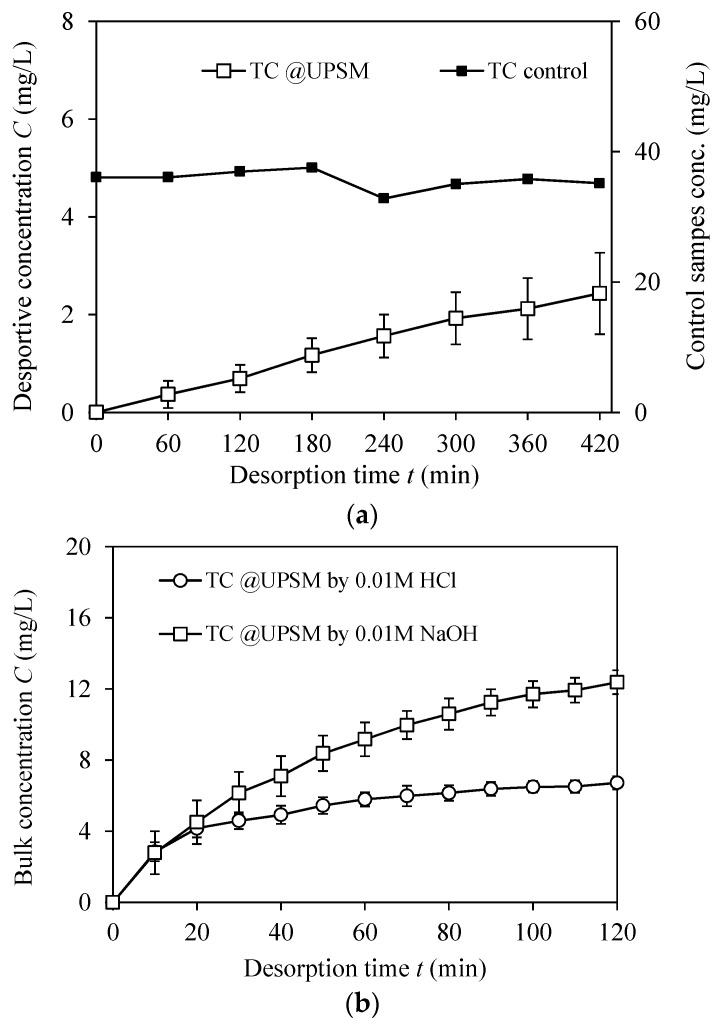
Desorption of recycling adsorbed antibiotics from loaded UPSM. (**a**) TC desorption in pure water at 35 °C for 7 h and pure TC solution used as control; (**b**) TC desorption by 0.01 M NaOH and 0.01 M HCl for 2 h. Error bars represent data deviation of duplicate experiments.

A desorption curve of TC from loaded UPSM by thermal treatment is shown in Figure 6a. The TC molecule was thermally stable at 35 °C according to the control curve. UPSM released 2 mg/L of TC in 7 h in a linear trend. According to Figure 6b, 12.4 mg/L of TC was released by 0.01 M HCl, which is higher than that by 0.01 M NaOH (6.7 mg/L). The mass of TC on UPSM was equal to 180 mg/L of TC in bulk solution, thus desorption rates of TC by heating, NaOH, and HCl in 2 h were 0.4%, 3.7%, and 6.9%, respectively.

The insufficient desorption by HCl indicated chemical association during the adsorption, such as π–π electron donor–acceptor (EDA) interaction and hydrogen bonding. Hydrogen bonding can be formed between the amino groups of tetracycline and the carbonyl groups of EDTA. The ketone group in tetracycline molecules is a π-electron acceptor and the ester group (–COO–R) in EDTA is a strong electron donor. Adsorption might be enhanced by different couples of π–π EDA interactions, e.g., the interaction between the conjugated π-electron moiety, the cation-π bonding between the amino groups, and the π-electron rich structures on the solid surface.

### 3.4. Adsorption Mechanisms

#### 3.4.1. FT-IR Analysis after Adsorption

**Figure 7:**
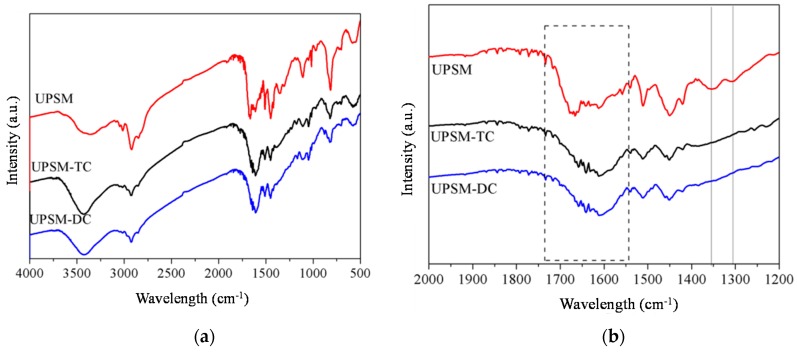
FT-IR spectra analysis of fresh, TC-loaded, and DC-loaded UPSM. (**a**) Full-spectrum curves; (**b**) zoomed spectra for carboxyl and hydrogen bonding.

After adsorption, specific peaks of pure tetracycline (Supplementary Figure S7) in the range of 3000–3500 cm^−1^ appeared in the FT-IR spectra of loaded UPSM (Figure 7a). The peaks at 1354 and 1305 cm^−1^ disappeared and the peak at 1650 cm^−1^ was blue shifted to about 1600 cm^−1^, which might be related to strong intermolecular hydrogen interactions between urea and tetracycline (Figure 7b).

#### 3.4.2. Role of Urea for Multilayer Adsorption

**Figure 8:**
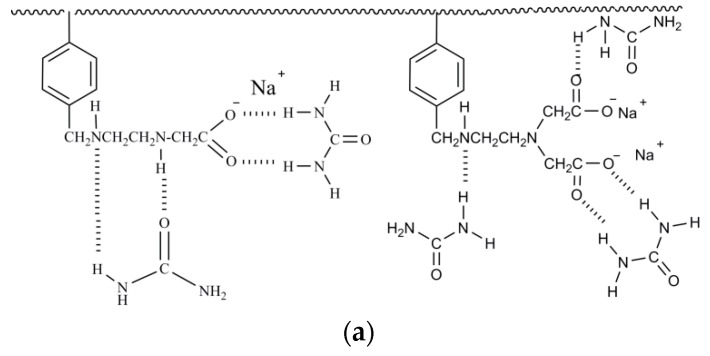

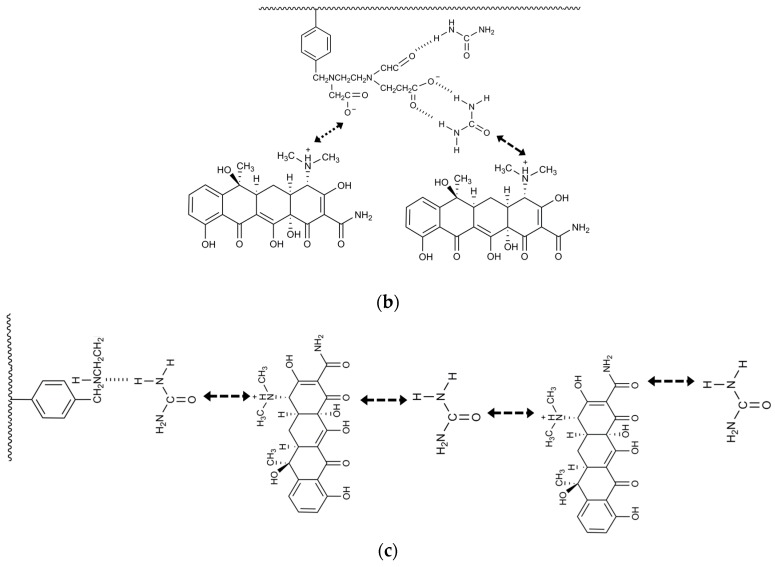
Proposed intermolecular hydrogen bond net corresponding to urea immersion and TC attraction. (**a**) Hydrogen bonds between urea and EDTA on PSM; (**b**) hydrogen bonds between tetracycline and urea or EDTA; (**c**) hydrogen bonds between urea and tetracycline to support multilayer adsorption.

Figure 8 shows the concepts of the bridging mechanism by urea immersion. During urea immersion, the ketone and amino groups of urea interact with the amino group and oxygen atoms of EDTA on the surface of PSM (Figure 8a). During the adsorption process, ketone groups of both urea and EDTA act as hydrogen acceptors to form hydrogen bonding (C–H…O) with carbonyl groups in tetracycline molecules (Figure 8b). During cycle urea immersion, two or more TC molecules can be linked due to their interaction with the urea molecules (Figure 8c). It is difficult for the tetracycline molecules to interact with each other directly due to their structural shape. However, the small-sized urea molecules acted as a lubricant to reduce such structural incompatibility, making the multilayer adsorption possible and feasible.

## 4. Conclusions

Urea immersion was used to modify microsphere resin to enhance its capacity of removing tetracycline reagents from water. The surface characteristics by XPS and FT-IR analysis confirmed successful urea immersion and tetracycline adsorption. The adsorption isotherms were explained by the Langmuir, Freundlich, and Tempkin models. The adsorption capacity of UPSM for TC was 460 mg/g and for DC was 430 mg/g, which were increased by 30% and 60%, respectively, by urea immersion. Adsorptive kinetic data were interpreted by first-order, second-order, and Weber–Morris models. The rate constant for TC adsorption on UPSM was 0.41/h and for DC was 0.33/h, indicating that the durations of 2.1 and 1.7 h were necessary for 50% removal of TC and DC, respectively.

Desorption experiments revealed pure dissociation of TC from UPSM. The releasing rates were 0.4%, 3.7%, and 6.9% in 2 h by heating, NaOH, and HCl, respectively. Possible chemical bonding such as hydrogen bonding and π–π interaction may contribute to the adsorption enhancement. Using urea molecules as bridges, multilayer adsorption of tetracycline was possible, which was confirmed by repeated adsorption experiments on UPSM with cycle urea immersion. The adsorption capacity was two to three times higher than the UPSM with single urea immersion. In summary, urea immersion is satisfactory to modify the surface of microsphere resin to enhance the removal of tetracycline antibiotics from water.

## Figures and Tables

**Table 1:** Data interpretation models for adsorption isotherms and kinetics.

Model Name	Equation	Lineweaver–Burk Equation	Coefficients
Langmuir	*q*_e_ = *q*_m_*·C*_e_/(*K*_L_ + *C*_e_)	*C*_e_/*q*_e_ = *C*_e_/*q*_m_ + *K*_L_/*q*_m_	*K*_L_, *q*_m_
Freundlich	*q*_e_ = *K*_f_*·C*_e_^1/*n*^	ln*q*_e_ = ln *K*_f_ + 1/*n·*ln*C*_e_	*K*_f_, *n*
Tempkin	*q*_e_ = RT/*b·*ln(*a·C*_e_)	*q*_e_ = RT/*b·*ln*a* + RT/*b·*ln*C*_e_	*a*, RT/*b*
First-order	d*q*_t_/d*t* = *K*_1_*·*(*q*_e_ − *q*_t_)	ln(*q*_e_ − *q*_t_) = ln*q*_e_ − *K*_1_*·t*	*K*_1_
Second-order	d*q*_t_/d*t* = *K*_2_*·*(*q*_e_ − *q*_t_)^2^	1*/t* = (*K*_2_*·q*_e_^2^) (1/*q*_t_ − 1/*q*_e_)	*K*_2_
Weber–Morris	*q*_t_ = *q*_e_*·K*_w_*·t*^1/2^	*q*_t_/*q*_e_ = *K*_w_*·t*^1/2^	*K*_w_

**Note**: *C*_e_ (mg/L) is the concentration of TC or DC at equilibrium in the solution, *q*_e_ (mg/g) is the amount of TC and DC adsorbed per unit weight of the adsorbents. The constant *q*_m_ (mg/g) is the maximal adsorption capacity in the Langmuir equation, *K*_L_ is related to the energy of adsorption (L/mg). *K*_f_ and *n* are constants of the Freundlich equation, which relate to adsorption capacity and intensity, respectively. T is the temperature of the solution (°C), R is the molar gas constant equal to 8.314 J/(K·mol), *a* and *b* are parameters of the Tempkin model. Parameters *q*_e_ and *q*_t_ (mg/g) are the amounts of TC adsorbed at equilibrium time and at time *t* (h) in the adsorption process, respectively. *K*_1_ (/h), *K*_2_ (g/(mg h)), and *K*_w_ (/h^1/2^) are the rate constants for first-order, second-order, and Weber–Morris kinetics, respectively.

**Table 2:** Isothermal fitting for tetracycline hydrochloride (TC) and doxycycline (DC) on PSM and UPSM at pH 6.8 and T = 298 K.

Adsorbent	Adsorbate	Langmuir			Freundlich			Tempkin		
		*q*_m_	*K*_L_	R^2^	*K*_f_	*n*	R^2^	*a*	RT/*b*	R^2^
PSM	TC	290	180	0.960	15	2.4	0.996	0.06	59	0.952
	DC	330	290	0.923	9.4	2.1	0.983	0.14	83	0.919
UPSM	TC	460	120	0.966	25	2.3	0.955	0.06	57	0.871
	DC	430	56	0.985	35	2.5	0.940	0.47	67	0.830

**Table 3:** Global estimation of kinetics for tetracycline adsorption on UPSM.

Target	First Rate Constant *K*_1_	R^2^	Second Rate Constant *K*_2_*q*_e_^2^	R^2^	Weber–Morris Constant *K*_w_	R^2^
	/h		mg/g/h		/h^1/2^	
TC	0.41	0.942	1.8	0.939	0.64	0.963
DC	0.33	0.929	2.1	0.933	0.63	0.962

**Table 4:** Comparison of TC adsorption capacity in the literature.

New Adsorbent	*q*_m_, mg/g	Reference
Magnetic multiamine resins	117	[35]
Magnetic polystyrene resins	166	[29]
Magnetic polydopamine resins	152	[36]
Polystyrene microsphere/graphene oxide	198	[31]
Polymer resins/anion exchange group	355	[37]
Magnetic microsphere/graphene oxide nanosheet	714	[38]
Nanosheet-layered double hydroxide	98	[39]
TiO_2_ nanosheets	213	[40]
Magnetic polyacrylonitrile nanofiber mat	315	[32]
Amino-ferrous functionalized silica	188	[24]
La-impregnated silicates	303	[33]
Activated carbons from hazelnut shell	303	[34]
Urea functionalized polystyrene resins	460	This study

## Supplementary Materials

The following are available online at http://www.mdpi.com/1660-4601/15/7/1524/s1, Figure S1: Structural formula of Tetracycline HCl (TC), Doxycycline HCl (DC) and original PSM, Figure S2: SEM image of original PSM and urea-immersed UPSM. (a-c) microsphere PSM in different scales, (d) urea modified microsphere UPSM before adsorption, (e) UPSM after adsorption of tetracycline, (f) UPSM after adsorption of tetracycline. Scale bar represents 500 μm in (a,d), 50 μm in (b,e) and 5μm in (c,f), Figure S3: The BET surface of original PSM and urea-immersed UPSM, Figure S4: The pore structures of original PSM and urea-immersed UPSM, Figure S5: Optimization of the initial pH value for tetracycline adsorption by comparing their kinetic curves. (a) Kinetic curves of TC on UPSM; (b) Kinetic curves of DC on UPSM; C_0_ is 60 mg/L, mass of UPSM is 60 mg, Figure S6: The XPS spectra analysis of fresh PSM and UPSM. (a) C 1s at PSM, (b) N 1s at PSM, (c) C 1s at UPSM and (d) N 1s at UPSM, , Figure S7: FT-IR spectra of TC and DC, Table S1: The molecular information of chemicals used in this study, Table S2: Porous structure information of the microspheres, Table S3: The kinetic parameters of tetracycline adsorption at different initial concentrations.

## References

[1] Kulkarni P., Olson N., Raspanti G., Rosenberg Goldstein R., Gibbs S., Sapkota A., Sapkota A. (2017). Antibiotic Concentrations Decrease during Wastewater Treatment but Persist at Low Levels in Reclaimed Water. Int. J. Environ. Res. Public Health.

[2] Gao P., Mao D., Luo Y., Wang L., Xu B., Xu L. (2012). Occurrence of sulfonamide and tetracycline-resistant bacteria and resistance genes in aquaculture environment. Water Res..

[3] Hladicz A., Kittinger C., Zarfel G. (2017). Tigecycline Resistant *Klebsiella pneumoniae* Isolated from Austrian River Water. Int. J. Environ. Res. Public Health.

[4] Zhang H., Li X., Yang Q., Sun L., Yang X., Zhou M., Deng R., Bi L. (2017). Plant Growth, Antibiotic Uptake, and Prevalence of Antibiotic Resistance in an Endophytic System of Pakchoi under Antibiotic Exposure. Int. J. Environ. Res. Public Health.

[5] Ji L., Wan Y., Zheng S., Zhu D. (2011). Adsorption of tetracycline and sulfamethoxazole on crop residue-derived ashes: Implication for the relative importance of black carbon to soil sorption. Environ. Sci. Technol..

[6] Pereira J.H., Queirós D.B., Reis A.C., Nunes O.C., Borges M.T., Boaventura R.A., Vilar V.J. (2014). Process enhancement at near neutral pH of a homogeneous photo-Fenton reaction using ferricarboxylate complexes: Application to oxytetracycline degradation. Chem. Eng. J..

[7] López Peñalver J.J., Sánchez Polo M., Gómez Pacheco C.V., Rivera Utrilla J. (2010). Photodegradation of tetracyclines in aqueous solution by using UV and UV/H_2_O_2_ oxidation processes. J. Technol. Biotechnol..

[8] Shi Y., Wang X., Qi Z., Diao M., Gao M., Xing S., Wang S., Zhao X. (2011). Sorption and biodegradation of tetracycline by nitrifying granules and the toxicity of tetracycline on granules. J. Hazard. Mater..

[9] Dirany A., Sirés I., Oturan N., Özcan A., Oturan M.A. (2012). Electrochemical treatment of the antibiotic sulfachloropyridazine: Kinetics, reaction pathways, and toxicity evolution. Environ. Sci. Technol..

[10] Chai R., Huang L., Li L., Gielen G., Wang H., Zhang Y. (2016). Degradation of Tetracyclines in Pig Manure by Composting with Rice Straw. Int. J. Environ. Res. Public Health.

[11] Kovalova L., Siegrist H., Singer H., Wittmer A., McArdell C.S. (2012). Hospital wastewater treatment by membrane bioreactor: Performance and efficiency for organic micropollutant elimination. Environ. Sci. Technol..

[12] Khamparia S., Jaspal D.K. (2017). Adsorption in combination with ozonation for the treatment of textile waste water: A critical review. Front. Environ. Sci. Eng..

[13] Acosta R., Fierro V., de Yuso A.M., Nabarlatz D., Celzard A. (2016). Tetracycline adsorption onto activated carbons produced by KOH activation of tyre pyrolysis char. Chemosphere.

[14] Shan D., Deng S., Zhao T., Wang B., Wang Y., Huang J., Yu G., Winglee J., Wiesner M.R. (2016). Preparation of ultrafine magnetic biochar and activated carbon for pharmaceutical adsorption and subsequent degradation by ball milling. J. Hazard. Mater..

[15] Parolo M.E., Savini M.C., Vallés J.M., Baschini M.T., Avena M.J. (2008). Tetracycline adsorption on montmorillonite: pH and ionic strength effects. Appl. Clay Sci..

[16] Zhang Z., Liu H., Wu L., Lan H., Qu J. (2015). Preparation of amino-Fe (III) functionalized mesoporous silica for synergistic adsorption of tetracycline and copper. Chemosphere.

[17] Turku I., Sainio T., Paatero E. (2007). Thermodynamics of tetracycline adsorption on silica. Environ. Chem. Lett..

[18] Yang W., Zheng F., Lu Y., Xue X., Li N. (2011). Adsorption interaction of tetracyclines with porous synthetic resins. Ind. Eng. Chem. Res..

[19] Zhou Q., Zhang M.C., Shuang C.D., Li Z.Q., Li A.M. (2012). Preparation of a novel magnetic powder resin for the rapid removal of tetracycline in the aquatic environment. Chin. Chem. Lett..

[20] Chao Y., Zhu W., Ye Z., Wu P., Wei N., Wu X., Li H. (2015). Preparation of metal ions impregnated polystyrene resins for adsorption of antibiotics contaminants in aquatic environment. J. Appl. Polym. Sci..

[21] Ma Y., Zhou Q., Li A., Shuang C., Shi Q., Zhang M. (2014). Preparation of a novel magnetic microporous adsorbent and its adsorption behavior of *p*-nitrophenol and chlorotetracycline. Hazard. Mater..

[22] Chao Y., Zhu W., Yan B., Lin Y., Xun S., Ji H., Wu X., Li H., Han C. (2014). Macroporous polystyrene resins as adsorbents for the removal of tetracycline antibiotics from an aquatic environment. J. Appl. Polym. Sci..

[23] Hao R., Xiao X., Zuo X., Nan J., Zhang W. (2012). Efficient adsorption and visible-light photocatalytic degradation of tetracycline hydrochloride using mesoporous BiOI microspheres. J. Hazard. Mater..

[24] Zhang Z., Li H., Liu H. (2018). Insight into the adsorption of tetracycline onto amino and amino-Fe3+ gunctionalized mesoporous silica: Effect of functionalized groups. J. Environ. Sci..

[25] Lv J., Ma Y., Chang X., Fan S. (2015). Removal and removing mechanism of tetracycline residue from aqueous solution by using Cu-13X. Chem. Eng. J..

[26] Yang L., Li Y., Wang L., Zhang Y., Ma X., Ye Z. (2010). Preparation and adsorption performance of a novel bipolar PS-EDTA resin in aqueous phase. J. Hazard. Mater..

[27] Li X., Yang L., Li Y., Ye Z., He A. (2012). Efficient Removal of Cd^2+^ from Aqueous Solutions by Adsorption on PS-EDTA Resins: Equilibrium, Isotherms, and Kinetic Studies. J. Environ. Eng. ASCE.

[28] Wang L., Yang L., Li Y., Zhang Y., Ma X., Ye Z. (2010). Study on adsorption mechanism of Pb(II) and Cu(II) in aqueous solution using PS-EDTA resin. Chem. Eng. J..

[29] Li B., Ma J., Zhou L., Qiu Y. (2017). Magnetic microsphere to remove tetracycline from water: Adsorption, H_2_O_2_ oxidation and regeneration. Chem. Eng. J..

[30] He J., Dai J., Xie A., Tian S., Chang Z., Yan Y., Huo P. (2016). Preparation of macroscopic spherical porous carbons@carboxymethylcellulose sodium gel beads and application for removal of tetracycline. RSC Adv..

[31] Chen L., Lei S., Wang M., Yang J., Ge X. (2016). Fabrication of macroporous polystyrene/graphene oxide composite monolith and its adsorption property for tetracycline. Chin. Chem. Lett..

[32] Liu Q., Zheng Y., Zhong L., Cheng X. (2015). Removal of tetracycline from aqueous solution by a Fe_3_O_4_ incorporated PAN electrospun nanofiber mat. J. Environ. Sci..

[33] Vu B.K., Snisarenko O., Lee H.S., Shin E.W. (2010). Adsorption of tetracycline on La-impregnated MCM-41 materials. Environ. Technol..

[34] Fan H., Shi L., Shen H., Chen X., Xie K. (2016). Equilibrium, isotherm, kinetic and thermodynamic studies for removal of tetracycline antibiotics by adsorption onto hazelnut shell derived activated carbons from aqueous media. RSC Adv..

[35] Zhu Z., Zhang M., Wang W., Zhou Q., Liu F. (2018). Efficient and synergistic removal of tetracycline and Cu(II) using novel magnetic multi-amine resins. Sci. Rep..

[36] Mao B., An Q., Xiao Z., Zhai S. (2017). Hydrophilic, hollow Fe_3_O_4_@PDA spheres with a storage cavity for efficient removal of polycyclic structured tetracycline. New J. Chem..

[37] Zhou Q., Wang M., Li A., Shuang C., Zhang M., Liu X., Wu L. (2013). Preparation of a novel anion exchange group modified hyper-crosslinked resin for the effective adsorption of both tetracycline and humic acid. Front. Environ. Sci. Eng..

[38] Hu X., Zhao Y., Wang H., Tan X., Yang Y., Liu Y. (2017). Efficient Removal of Tetracycline from Aqueous Media with a Fe_3_O_4_ Nanoparticles@graphene Oxide Nanosheets Assembly. Int. J. Environ. Res. Public Health.

[39] Soori M.M., Ghahramani E., Kazemian H., Al-Musawi T.J., Zarrabi M. (2016). Intercalation of tetracycline in nano sheet layered double hydroxide: An insight into UV/VIS spectra analysis. J. Taiwan Inst. Chem. Eng..

[40] Fu D., Huang Y., Zhang X., Kurniawan T.A., Ouyang T. (2017). Uncovering potentials of integrated TiO_2_(B) nanosheets and H_2_O_2_ for removal of tetracycline from aqueous solution. J. Mol. Liq..

[41] Borghi A.A., Palma M.S.A. (2014). Tetracycline: Production, waste treatment and environmental impact assessment. Braz. J. Pharm. Sci..

