# Supplementary material for: Simple Urea Immersion Enhanced Removal of Tetracycline from Water by Polystyrene Microspheres
Source: Int J Environ Res Public Health. 2018 Jul 19;15(7):1524. doi: 10.3390/ijerph15071524 (PMC6068535; doi:10.3390/ijerph15071524)
Supplement: Supplementary file 1 [file ijerph-15-01524-s001.pdf]

Simple Urea Immersion Enhanced Removal of Tetracycline from Water by Polystyrene Microspheres

Junjun Ma, Bing Li, Lincheng Zhou, Yin Zhu, Ji Li and Yong Qiu

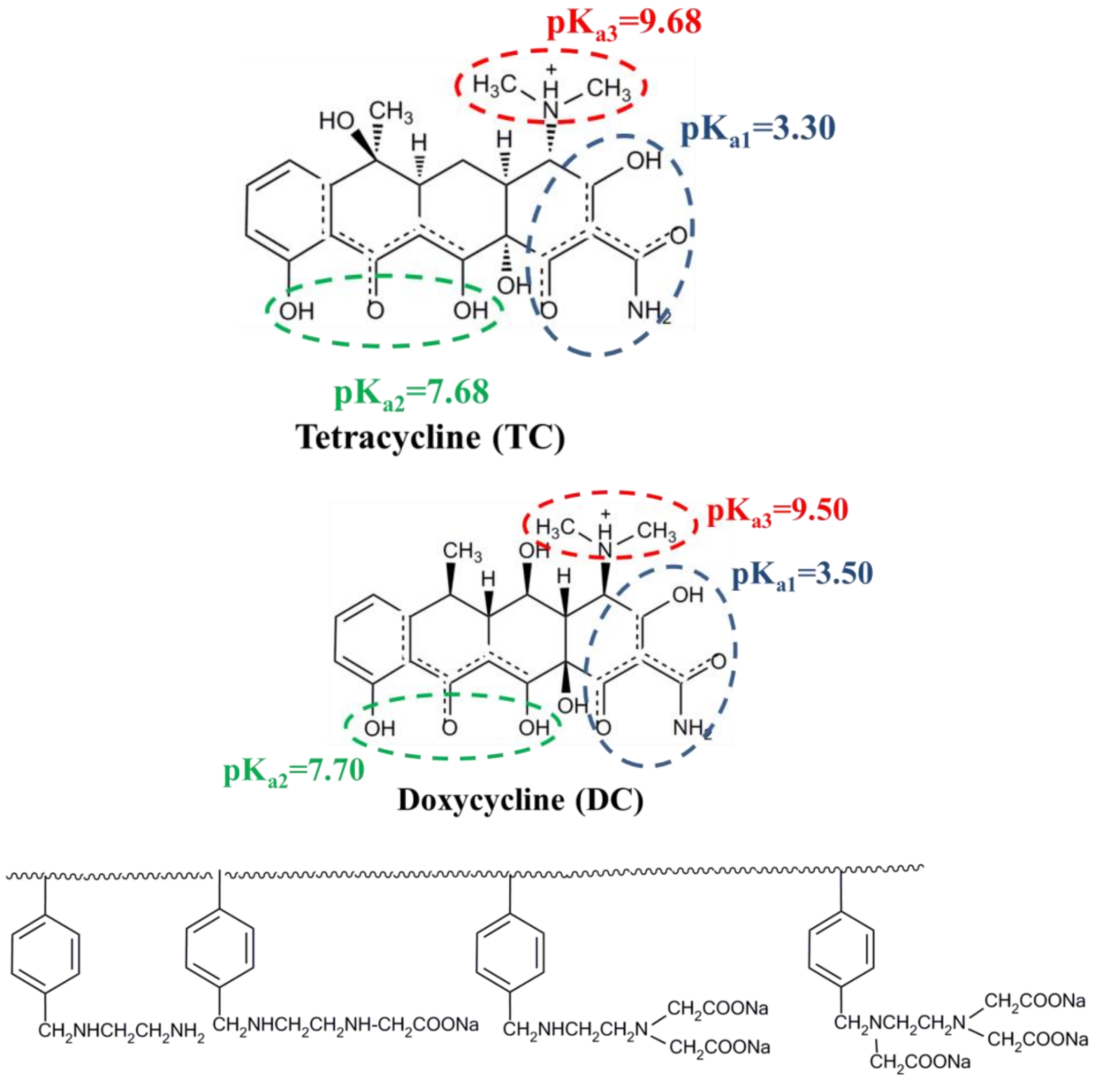

Figure S1. Structural formula of Tetracycline HCl (TC), Doxycycline HCl (DC) and original PSM.

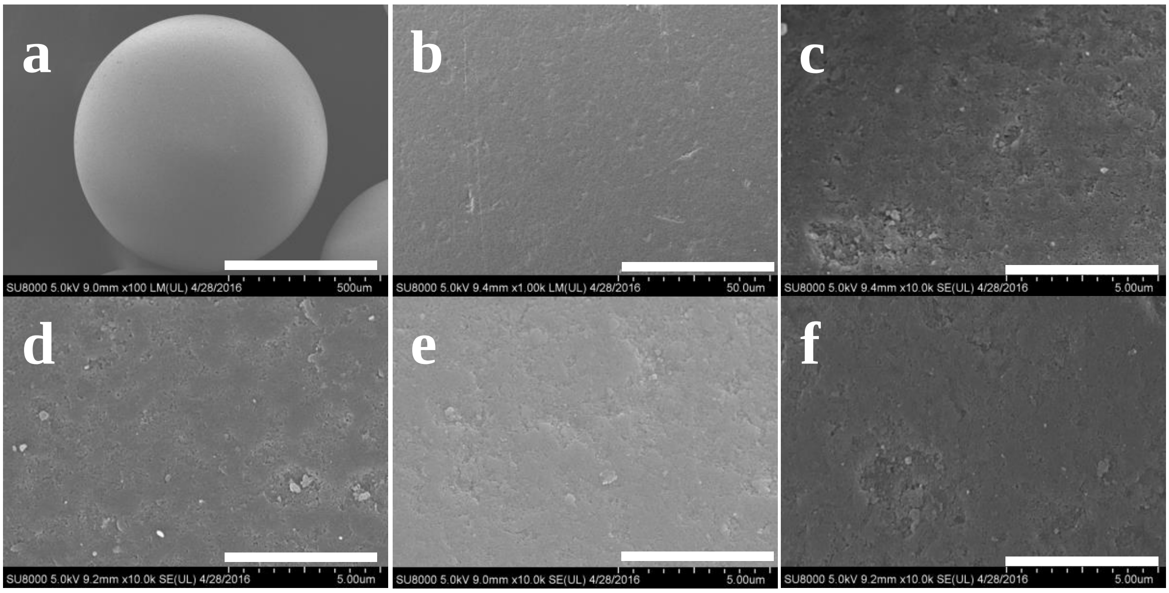

Figure S2. SEM images of original PSM and urea-immersed UPSM. (a-c) microsphere PSM in different scales, (d) urea modified microsphere UPSM before adsorption, (e) UPSM after adsorption of tetracycline, (f) UPSM after adsorption of tetracycline. Scale bar represents 500 μm in (a,d), 50 μm in (b,e) and 5μm in (c,f).

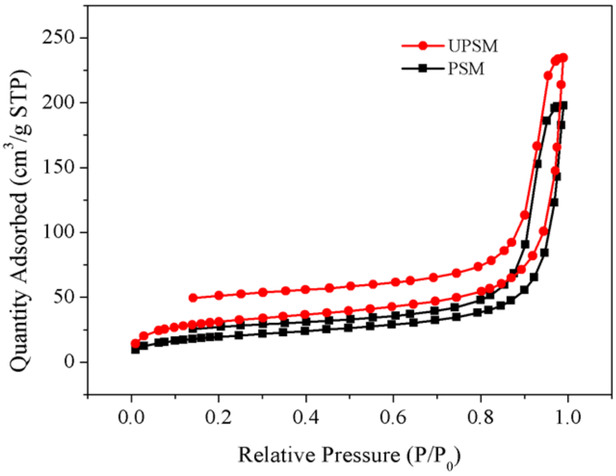

Figure S3. The BET surface of original PSM and urea-immersed UPSM.

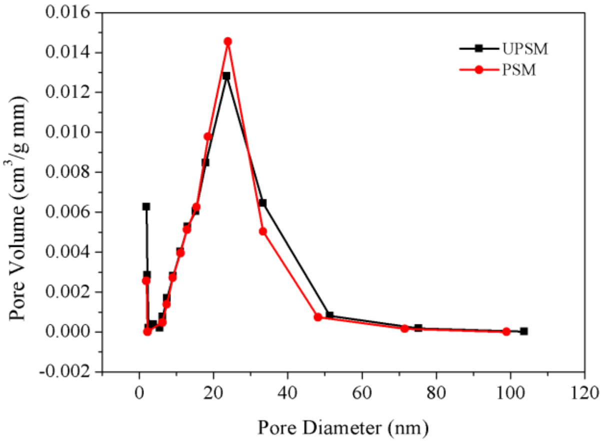

Figure S4. The pore structures of original PSM and urea-immersed UPSM.

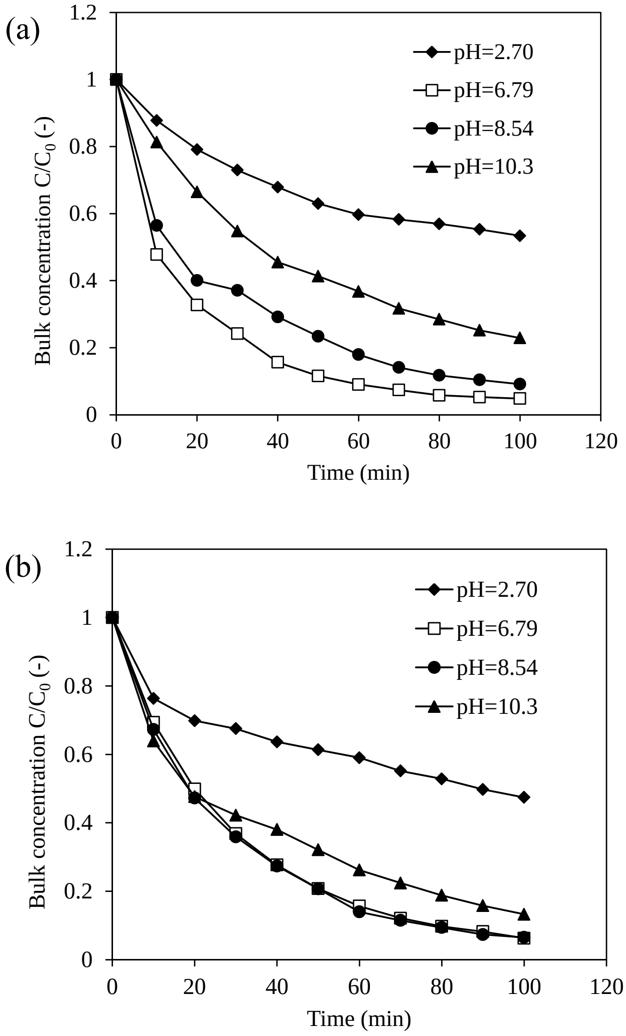

Figure S5. Optimization of the initial pH value for tetracycline adsorption by comparing their kinetic curves. (a) Kinetic curves of TC on UPSM; (b) Kinetic curves of DC on UPSM; C_0 is 60 mg/L, mass of UPSM is 60 mg.

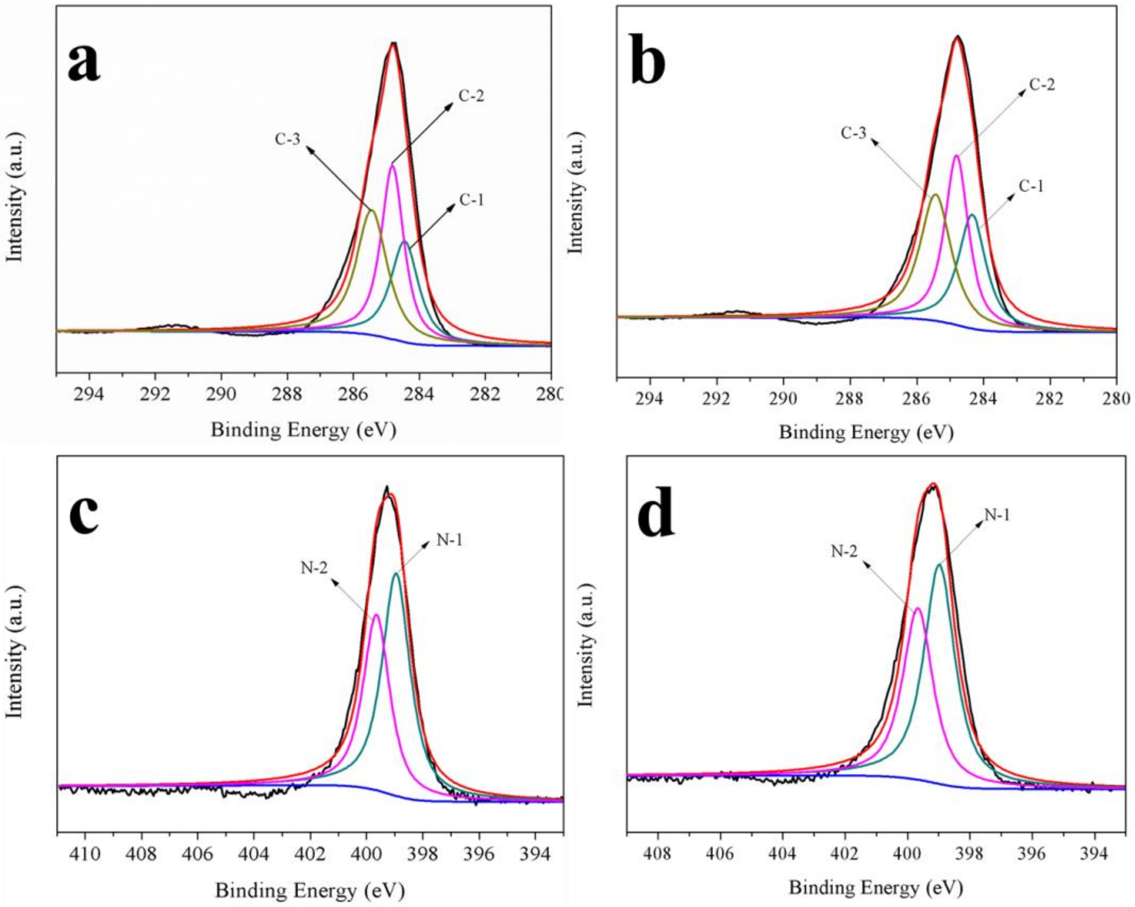

Figure S6. The XPS spectra analysis of fresh PSM and UPSM. (a) C 1s at PSM, (b) N 1s at PSM, (c) C 1s at UPSM and (d) N 1s at UPSM.

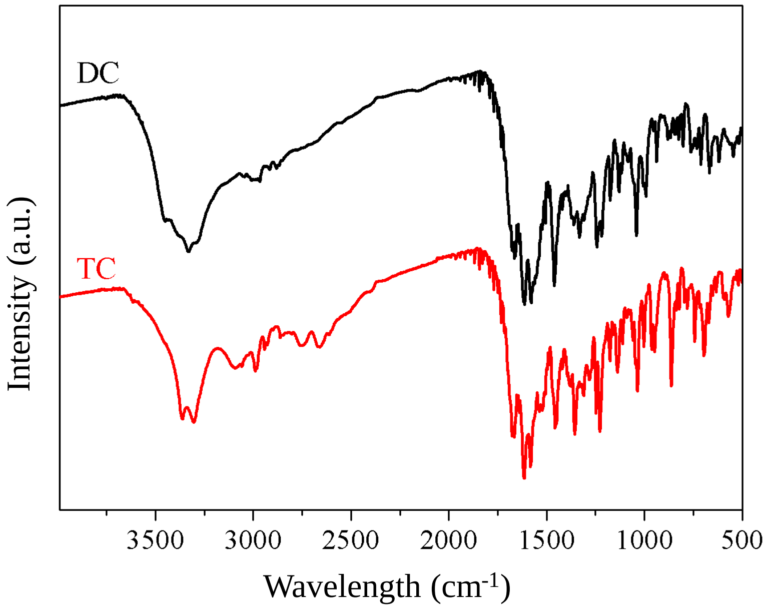

Figure S7. FT-IR spectra of TC and DC.

Table S1. The molecular information of chemicals used in this study.

Name	CAS number	Formula	Molar weight
Urea	57-13-6	CH_4N_2O	60.06
Tetracycline hydrochloride	64-75-5	$C_{22}H_{26}ClN_2O_9$	480.90
Doxycycline hydrochloride	24390-14-5	$C_{22}H_{25}ClN_2O_8$	462.45

Table S2. Porous structure information of the microspheres.

Microsphere	surface area	Pore Volume	Pore size
**Unit**	**m^2/g**	**cm^3/g**	**nm**
PSM	71.69	0.3	22.6
UPSM	112.4	0.34	20.4

Table S3. The kinetic parameters of tetracycline adsorption at different initial concentrations.

	Initial concen. C_0 mg/L	1st order rate constant, K_1 /h	R^2	2nd order rate constant, $K_2q_e^2$ mg/g/h	R^2	Weber-Morris model constant, K_w /$h^{1/2}$	R^2
TC @UPSM	100	0.31	0.865	2.2	0.871	0.84	0.996
	150	0.42	0.965	1.9	0.944	0.77	0.992
	200	0.43	0.979	1.7	0.996	0.70	0.983
	250	0.43	0.992	1.5	0.998	0.65	0.965
	300	0.44	0.996	1.4	0.997	0.61	0.947
DC @UPSM	100	0.31	0.838	1.2	0.984	0.85	0.993
	150	0.32	0.895	1.5	0.995	0.83	0.989
	200	0.33	0.953	1.8	0.997	0.74	0.976
	250	0.34	0.976	1.3	1.000	0.73	0.956
	300	0.35	0.985	1.4	1.000	0.70	0.943
TC @PSM	50	0.37	0.907	0.8	0.954	0.62	0.988
	100	0.40	0.875	3.0	0.864	0.67	0.995
	150	0.41	0.973	1.3	0.975	0.53	0.995
	200	0.42	0.987	1.3	0.984	0.50	0.994
	250	0.43	0.996	1.0	0.994	0.45	0.991
